# Pseudonormokalemia case report - What does it mean to have normal blood potassium?

**DOI:** 10.11613/BM.2024.021002

**Published:** 2024-06-15

**Authors:** Tomáš Šálek, David Stejskal

**Affiliations:** 1 Institute of Laboratory Medicine, Medical Faculty, University of Ostrava, Ostrava, Czechia; 2 Department of Clinical Biochemistry and Pharmacology, The Tomas Bata Hospital in Zlín, Zlín, Czechia

**Keywords:** pseudonormokalemia, hypokalemia, interference, thrombocytosis

## Abstract

This case report describes a case of pseudonormokalemia, true hypokalemia. Often, only laboratory values outside the normal range gain attention and false normal results are at risk of not being noticed. However, a disease state may be masked by another pathological process. Here, a 50-year old male was admitted to the Department of Internal Medicine due to sepsis from a dental infection. Initially, serum potassium measurement revealed a normal value of 4 mmol/L (reference interval 3.8-5.1 mmol/L). Thrombocyte number was above 500x10^9^/L. Due to our policy to recommend a repeated measurement of potassium in whole blood or heparin plasma if a patient has thrombocytosis, pseudonormokalemia was identified because the heparin plasma potassium value was only 2.9 mmol/L (reference interval 3.5-4.8 mmol/L). The physiological difference between serum and plasma concentration is no more than 0.3 mmol/L. In this case, potassium concentration were falsely elevated in the serum sample, probably caused by the high number of platelets releasing potassium during clotting. Interpretative comments in patients with thrombocytosis over 500x10^9^/L recommending plasma potassium measurement are helpful. The best way to eliminate pseudohyperkalemia and pseudonormokalemia phenomena caused by thrombocytosis is to completely change towards heparin plasma as the standard material.

## Graphical abstract

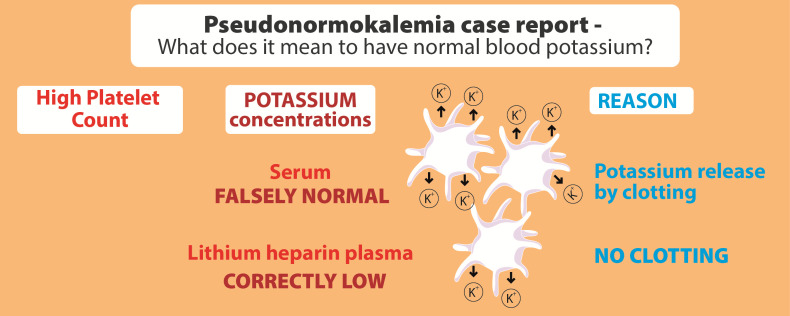

## Highlights

• Pseudonormokalemia may mean thrombocytosis, serum potassium concentration within reference ranges, and low plasma potassium concentration

• The best way to eliminate both pseudohyperkalemia and pseudonormokalemia phenomenon is to completely change towards heparin-plasma as the standard material

## Introduction

The total testing process starts with the clinical question, followed by test selection, order, sample collection, sample transportation, test measurement, result reporting, clinical answer and clinical action. It finishes with the effect of patient care (*1*). The physician’s attention is logical when the test result is out of reference ranges or exceeds decision values. Considering a pathological state when the test results are falsely normal is more difficult. The intracellular potassium concentration is about 140-150 mmol/L, while plasma physiological concentration is 3.5-5.0 mmol/L (*2*). Potassium can be released from blood cells during sample collection, transportation, clotting and centrifugation. The most common cause of potassium leakage from blood cells is hemolysis. It may result from inappropriate collection needles, intravenous catheter blood collection, syringe draw, traumatic draw, extended tourniquet time, vigorous mixing of tubes, agitation during transport and underfilling (*3*). Automated analyzers measure the hemolysis index in all serum/plasma laboratory samples. Visual assessment of hemolysis is not recommended and can seriously jeopardize patient safety (*4*). Falsely elevated blood potassium may also be caused by pumping with the fist, low-temperature whole blood storage, long storage times, hereditary pseudohyperkalemia, thrombocytosis, leukocytosis, *etc.* (*3*). It is a challenge for laboratories to detect falsely elevated potassium results if it is not caused by hemolysis.

Missed and left untreated pseudonormokalemia could lead to dangerous situations resulting from hypokalemia, such as cardiac arrhythmias (*5*).

This case study aims to present a patient with pseudonormokalemia, true hypokalemia. A 50-year-old male was admitted to the Department of Internal Medicine due to sepsis from a dental infection. He had Addison‘s disease, hypothyroidism, asthma, depression and penicillin allergy. His regular medication included Hydrocortisone 10 mg 2-1-0, Levothyroxine 50 µg 1.5-0-0, Mirtazapine 45 mg 0-0-0-1, and budesonide-formoterol inhalation. Upon admission, a physical examination revealed a blood pressure of 115/70, a regular heart rate of 110/min, vesicular breathing, a body temperature of 38.9 °C and a body mass index of 29.6 kg/m^2^. The basic biochemistry tests, including electrolytes, were collected.

The patient signed informed consent for the publication of his case study. The publication was approved by the local Tomas Bata Hospital Ethics Committee.

## Laboratory analyses

The basic biochemistry tests during antibiotic treatment showed normokalemia. The blood collection was performed in the morning at 6.00 and serum indices showed no hemolysis (free hemoglobin was 0.00 g/L), lipemia or icterus. The blood count revealed thrombocytosis and anemia. All tests are displayed in Table 1.

**Table 1:** Serum laboratory tests and blood count (collection at 6.00)

**Laboratory test**	**Result**	**Reference interval**
Na (mmol/L)	139	136-144
K (mmol/L)	4.0	3.8-5.1
Cl (mmol/L)	108	95-107
Urea (mmol/L)	6.7	3.0-8.0
CREA (µmol/L)	62	49-90
eGFR - CKD-EPI from serum creatinine (mL/min/1.73m^2^)	78	90-150
Cystatin C (mg/L)	1.41	< 0.96
eGFR - CKD-EPI from cystatin C (mL/min/1.73m^2^)	51	90-150
TBIL (µmol/L)	15	< 20
ALT (U/L)	157.8	< 43.8
Alb (g/L)	46.0	36.0-45.0
CRP (mg/L)	10.0	0.0-2.0
WBC (EDTA sample) (x10^9^/L)	9.8	4.0-10.0
Hb (EDTA sample) (g/L)	112	135-175
Plt (EDTA sample) (x10^9^/L)	571	150-400
Na - sodium. K - potassium. Cl - chloride. CREA - creatinine. eGFR - estimated glomerular filtration rate. CKD-EPI - chronic kidney disease epidemiology collaboration. TBIL - total bilirubin. ALT - alanine aminotransferase. Alb - albumin. CRP - C-reactive protein. WBC - white blood cells. Hb - hemoglobin. Plt - platelets.		

The serum sample was drawn in a 6 mL VACUETTE red top tube with clot aktivator (Greiner Bio-One Gmbh, catalog number 476092, Kremsmunster, Austria). The 10-minute centrifugation at 1500xg was done within one hour after sampling. Our laboratory performs two checks when reporting laboratory test results. Biomedical scientists perform the technical validation, which includes evaluating the impact of serum indices and other preanalytical factors on laboratory test results. The medical validation is performed by specialists in laboratory medicine and it covers the consideration of the clinical plausibility of all results. If a patient has thrombocytosis over 500x10^9^/L, pseudohyperkalemia or pseudonormokalemia is considered (*6*). The plasma potassium measurement in whole blood or a lithium heparin tube is recommended by the written interpretative comment on the result report.

## Further investigation

The lithium heparin plasma sample was collected at 9.40, the results were available in 51 minutes and revealed hypokalemia. All plasma results are shown in Table 2.

**Table 2:** Plasma laboratory tests (lithium heparin tube, collection at 9.40)

**Laboratory test**	**Result**	**Reference interval**
Na (mmol/L)	137	136-144
K (mmol/L)	2.9	3.5-4.8
Cl (mmol/L)	106	95-107
Mg (mmol/L)	0.83	0.80-0.94
Na - sodium. K - potassium. Cl - chloride. Mg - magnesium.		

The plasma sample was measured in a 3 mL green top lithium heparin tube from the same manufacturer (catalog number 454082). The same centrifugation conditions were used as for serum sample. All biochemical tests were measured on Abbott Architect analyzer ci 16200 (Abbott Laboratories, Illinois, USA). This situation can be concluded as pseudonormokalemia. The thrombocytosis was temporary. After the successful antibiotic treatment of sepsis, the thrombocyte count was 242x10^9^/L and serum potassium concentration was 4.2 mmol/L. Results before hospital discharge are shown in Table 3. The thrombocyte count decreased due to the successful treatment of inflammation and potassium concentration increased after substitution therapy.

**Table 3:** Results before hospital discharge (collection at 6.00, serum sample)

**Laboratory test**	**Result**	**Reference interval**
Na (mmol/L)	142	136-144
K (mmol/L)	4.2	3.8-5.1
Cl (mmol/L)	111	95-107
Urea (mmol/L)	6.0	3.0-8.0
CREA (µmol/L)	85	49-90
eGFR (CKD-EPI equation) from serum creatinine (mL/min/1.73m^2^)	92	90-150
Glc (mmol/L)	4.8	3.9-5.5
CRP (mg/L)	2.0	0.0-2.0
WBC (EDTA sample) (x10^9^/L)	8.3	4.0-10.0
Hb (EDTA sample) g/L	125	135-175
Plt (EDTA sample) (x10^9^/L)	242	150-400
Na - sodium. K - potassium. Cl - chloride. CREA - creatinine. eGFR - estimated glomerular filtration rate. CKD-EPI - chronic kidney disease epidemiology collaboration. Glc - glucose. CRP - C-reactive protein. WBC - white blood cells. Hb - hemoglobin. Plt - platelets.		

## What happened?

The patient initially had thrombocytosis and normal serum potassium concentrations. Results are shown in Table 1. Plasma potassium measurement revealed hypokalemia of 2.9 mmol/L. All results are visible in Table 2.

We have seen the difference between serum and plasma potassium concentrations of 1.1 mmol/L.

In this case, the elevated serum potassium may result from its release from platelets and other cells during clotting. The impact of high platelet numbers on potassium results is only seen in serum, and falsely elevated potassium results can be identified by comparing the results to heparin plasma values.

## Discussion

This case study describes a case study of pseudonormokalemia, true hypokalemia, probably due to thrombocytosis. The physiological difference in potassium concentration between plasma and serum is no more than 0.3 mmol/L (*7*). The cut-off for pseudohyperkalemia, the difference between serum and plasma concentrations, was defined as 1.0 mmol/L (*8*). Whole blood was suggested for plasma potassium measurement on blood gas analyzers (*8*). On these instruments, the undetectable hemolysis is a real potassium measurement problem, which is why plasma is the better choice. The hemolysis detection on these devices may be available in the future. Lithium-heparin plasma after centrifugation is another option; it is the easiest way to identify falsely elevated potassium results caused by thrombocytosis (*9*). The diagnosis of pseudonormokalemia is an even more significant challenge compared to pseudohyperkalemia. Delgado *et al.* reported that up to 0.14% of the total serum potassium determinations were susceptible to pseudohyperkalemia or pseudonormokalemia. Pseudonormokalemia accounted for 85% of cases (*10*). We can conclude that pseudonormokalemia was also presented in this case study.

## What YOU should / can do in your laboratory to prevent such errors

The best way to eliminate this phenomenon is to change towards heparin-plasma as the standard material completely. Interpretative comments in patients with thrombocytosis over 500x10^9^/L recommending plasma potassium measurement are helpful even in patients with normal serum potassium values. This comment may be added to all reports with thrombocyte results over 500x10^9^/L. It explains that the patient has probably falsely elevated serum potassium results and recommends the lithium heparin plasma sample collection for potassium measurement.

## Data Availability

All data generated and analyzed in the presented study are included in this article.

## References

[1] Schumacher GE Barr JT. Total testing process applied to therapeutic drug monitoring: impact on patients’ outcomes and economics. Clin Chem. 1998;44:370–4. 10.1093/clinchem/44.2.370 9474047

[2] Zacchia M Abategiovanni ML Stratigis S Capasso G. Potassium: From Physiology to Clinical Implications. Kidney Dis (Basel). 2016;2:72–9. 10.1159/000446268 27536695 PMC4947686

[3] Schlüter K Cadamuro J. Erroneous potassium results: preanalytical causes, detection, and corrective actions. Crit Rev Clin Lab Sci. 2023;60:442–65. 10.1080/10408363.2023.2195936 37042478

[4] Lippi G Cadamuro J. Visual assessment of sample quality: quo usque tandem? Clin Chem Lab Med. 2018;56:513–5. 10.1515/cclm-2017-0867 29055938

[5] Clase CM Carrero JJ Ellison DH Grams ME Hemmelgarn BR Jardine MJ Potassium homeostasis and management of dyskalemia in kidney diseases: conclusions from a Kidney Disease: Improving Global Outcomes (KDIGO) Controversies Conference. Kidney Int. 2020;97:42–61. 10.1016/j.kint.2019.09.018 31706619

[6] Thurlow V Ozevlat H Jones SA Bailey IR. Establishing a practical blood platelet threshold to avoid reporting spurious potassium results due to thrombocytosis. Ann Clin Biochem. 2005;42:196–9. 10.1258/0004563053857761 15949154

[7] Sevastos N Theodossiades G Archimandritis AJ. Pseudohyperkalemia in serum: a new insight into an old phenomenon. Clin Med Res. 2008;6:30–2. 10.3121/cmr.2008.739 18591376 PMC2442023

[8] Ranjitkar P Greene DN Baird GS Hoofnagle AN Mathias PC. Establishing evidence-based thresholds and laboratory practices to reduce inappropriate treatment of pseudohyperkalemia. Clin Biochem. 2017;50:663–9. 10.1016/j.clinbiochem.2017.03.007 28288853

[9] Šálek T. Pseudohyperkalemia - Potassium released from cells due to clotting and centrifugation - a case report. Biochem Med (Zagreb). 2018;28:011002. 10.11613/BM.2018.011002 29472808 PMC5806620

[10] Delgado JA Lopez B Morell-García D Martínez-Morillo E Antonieta Ballesteros M Jiménez SA Clinical Thresholds for Pseudohyperkalemia and Pseudonormokalemia in Patients with Thrombocytosis. EJIFCC. 2022;33:233–41. 36447798 PMC9644095

